# Tyramine Reveals Failing α_2_-Adrenoceptor Control of Catecholamine Release and Total Peripheral Vascular Resistance in Hypertensive Rats

**DOI:** 10.3389/fneur.2013.00019

**Published:** 2013-02-28

**Authors:** Torill Berg, Jørgen Jensen

**Affiliations:** ^1^Department of Physiology, Institute of Basic Medical Sciences, University of OsloOslo, Norway; ^2^Department of Physical Performance, Norwegian School of Sport SciencesOslo, Norway

**Keywords:** α_2_-adrenoceptors, hypertension, sympathetic nervous system, norepinephrine, epinephrine, release control, total peripheral vascular resistance

## Abstract

α_2_-Adrenoceptor-activation lowers central sympathetic output, peripheral, vesicular norepinephrine release, epinephrine secretion, and modulates vascular tension. We previously demonstrated that α_2_-adrenoceptor-mediated inhibition of basal norepinephrine release was not reflected in plasma unless re-uptake through the norepinephrine transporter (NET) was blocked. Tyramine activates reverse norepinephrine transport through NET. Here we tested the hypothesis that tyramine, by engaging NET in release, also blocks re-uptake, and therefore allows manipulation of pre-junctional α_2_-adrenoceptors to directly regulate norepinephrine overflow to plasma. We compared in anesthetized spontaneously hypertensive rats (SHRs) and normotensive controls (WKYs), the effect of α_2_-adrenoreceptor antagonist (L-659,066) and/or agonist (clonidine) on norepinephrine overflow and increase in total peripheral vascular resistance (TPR) evoked by tyramine-infusion (1.26 μmol/min/kg, 15 min) and epinephrine secretion activated by the surgical stress. TPR was computed as blood pressure divided by cardiac output, recorded as ascending aortic flow. Plasma catecholamine concentrations after tyramine were higher in SHRs than WKYs. Pre-treatment with L-659,066 increased the catecholamine concentrations in WKYs, but only if combined with clonidine in SHRs. Clonidine alone reduced tyramine-induced norepinephrine overflow in SHRs, and epinephrine in both strains. Tyramine-induced increase in TPR was not different after clonidine, eliminated after L-659,066 and L-659,066 + clonidine in WKYs, but only after L-659,066 + clonidine in SHRs. We conclude that tyramine-infusion does allow presynaptic regulation of vesicular release to be accurately assessed by measuring differences in plasma norepinephrine concentration. Our results indicate that presynaptic α_2_-adrenoceptor regulation of norepinephrine release from nerve vesicles and epinephrine secretion is dysfunctional in SHRs, but can be restored by clonidine.

## Introduction

**Figure 1:**
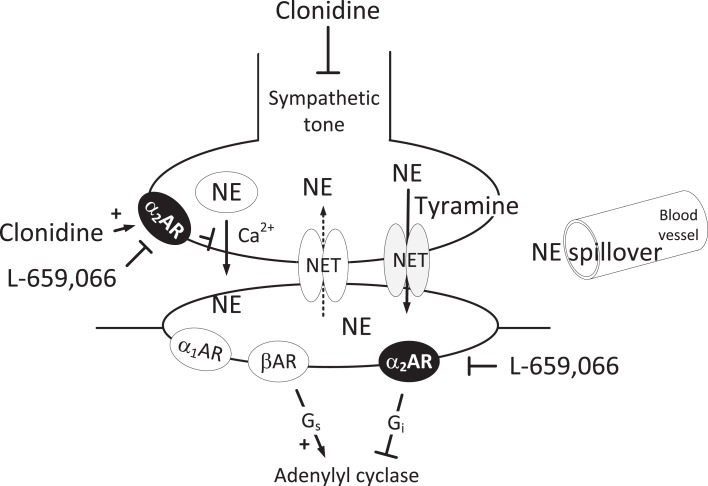
**Norepinephrine release in the sympathetic synapse**. Vesicular release is stimulated by Ca^2+^ and inhibited by α_2_AR. The re-uptake of norepinephrine through NET can be reversed to release by tyramine. Then NET will be engaged in release and re-uptake is hampered, and overflow to plasma is increased. However, vesicular norepinephrine release is not directly influenced by this process, and will still be subjected to presynaptic control by the α_2_AR or other presynaptic receptors. Clonidine will lower the sympathetic tone through a central action, as well as stimulate presynaptic and postsynaptic α_2_AR. L-659,066, which does not cross the blood-brain barrier, will inhibit the peripheral pre- and postsynaptic α_2_AR. In the VSMC, L-659,066 will allow βAR-G_s_-signaling to dominate tension control when norepinephrine release is stimulated. Blunted arrows indicate inhibition.

Through their ability to inhibit norepinephrine release, the α_2_-adrenoceptors (AR) represent a last line of defense against sympathetic hyperactivity. This is also true for the release of catecholamines from the adrenal medulla. The level of adrenergic activity is often assessed by the plasma concentration of catecholamines. In fact, epinephrine is directly released into plasma when the adrenal medulla is stimulated, therefore plasma epinephrine concentration reflects release. However, norepinephrine from sympathetic nerve endings is released into the synapse, where it activates postsynaptic α_1_AR, α_2_AR, and/or βAR, but some norepinephrine escapes these receptors and may modulate transmitter release by activating presynaptic AR. The signal is terminated by the return of norepinephrine from the synapse into the nerve varicosities by re-uptake through the norepinephrine transporter (NET; Figure 1). Only a fraction of the released norepinephrine therefore enters the circulation, and the plasma norepinephrine concentration does not directly reflect the amount released. In recent studies on anesthetized normotensive (WKY) rats under resting conditions, we demonstrated that inhibition of either NET or α_2_AR did not alter the plasma norepinephrine concentration. This observation indicated that one mechanism substituted for the other in limiting the concentration of norepinephrine in the synapse, and, hence, overflow to plasma (Berg et al., 2012). However, if we injected the α_2_AR antagonist after prior administration of the NET inhibitor desipramine, the plasma norepinephrine concentration increased. Therefore, presynaptic α_2_AR-mediated inhibition of release was not reflected as a change in the plasma norepinephrine concentration unless re-uptake was blocked. By contrast, in this same study, the augmenting effect of α_2_AR antagonist in desipramine-treated rats was far greater in spontaneously hypertensive rats (SHRs) than in WKYs (Berg et al., 2012).

The α_2_AR are divided into three subtypes, i.e., α_2A_, α_2B_, and α_2C_, and deletion of the α_2A_AR-gene created a hypertensive mouse with high plasma norepinephrine levels (Makaritsis et al., 1999). Elevated sympathetic tone plays an important role in initiating and sustaining hypertension in SHRs as in essential hypertension in human (Esler, 2010), and there is evidence that a failing α_2_AR inhibition of catecholamine release may contribute to the high sympathetic activity. Thus, impaired α_2_AR-mediated inhibition of release has been described in both the central nervous system (CNS) and in peripheral nerves of SHRs (Yamada et al., 1989; Remie et al., 1992; Reja et al., 2002; Zugck et al., 2003). Indeed, we demonstrated impaired CNS α_2_AR inhibition by using clonidine, which easily penetrates the blood-brain barrier. In the presence of the α_2_AR antagonist L-659,066, which does not cross the blood-brain barrier, clonidine normalized the elevated mean arterial blood pressure (MBP), heart rate (HR), and total peripheral vascular resistance (TPR) and reduced norepinephrine overflow to plasma in SHRs (Berg et al., 2012). However, in the resting condition, we did not detect a failing α_2_AR control of peripheral catecholamine release in SHRs (Berg et al., 2012).

α_2_AR on vascular smooth muscle cells (VSMC) induce vasoconstriction (Philipp et al., 2002), whereas endothelial α_2A_AR may activate nitric oxide (NO)-dependent vasodilatation (Shafaroudi et al., 2005). Vasoconstriction evoked by stimulation of α_2_AR on VSMC has been shown to be impaired in SHRs (Feres et al., 1998), and a vasodilatory α_2_AR-component, which opposed a sympathetic TPR-response in WKYs, was not observed in SHRs (Berg and Jensen, 2011). Activation of inhibitory G-proteins (G_i_) and inhibition of adenylyl cyclase is one mechanism by which α_2_AR may activate vasoconstriction, counter-balanced by the stimulatory effect of βAR on adenylyl cyclase (Figure 1). However, the moderating effect of α_2_AR antagonist on a phenylephrine-induced, α_1_AR-mediated vasoconstriction did not differ in the two strains (Berg et al., 2012). This may be different when endogenous norepinephrine is released into the synapse, and can activate not only α_1_- but also α_2_- and βAR. In addition, other signaling substances, such as neuropeptide Y and ATP, co-released with norepinephrine, may influence the response (Burnstock and Verkhratsky, 2010).

In order to demonstrate the full effect of α_2_AR stimulation on catecholamine release and TPR, *in vivo* experiments are required involving inhibition of re-uptake through NET and concomitant activation of endogenous norepinephrine release. Norepinephrine release can be activated by tyramine, which stimulates peripheral release by reverse transport through NET (Mandela and Ordway, 2006; Berg et al., 2010), and unlike vesicular release, NET-mediated release is not under presynaptic control (Starke, 2001). However, since tyramine will engage NET in release, we hypothesized that re-uptake is also prevented (Figure 1), and presynaptic modulation of vesicular release reflected as differences in the concomitant tyramine-stimulated norepinephrine overflow to plasma. This hypothesis was tested in the present study. We recorded BP and cardiac output in order to establish how α_2_AR activation influenced TPR during tyramine-infusion. Because we hypothesized that the function of α_2_AR is different in WKYs and SHRs, we carried out similar experiments on the two strains.

## Materials and Methods

### Experimental procedure

All experiments were approved by the institutional review committee, and conducted in accordance with the National Institutes of Health (NIH) Guide for the Care and Use of Laboratory Animals. Twelve- to fourteen-weeks-old, male WKYs (*n* = 57, 289 ± 5 g body weight) and SHRs (Okamoto, SHR/NHsd strain, *n* = 58, 284 ± 4 g body weight) on 12/12 h light/dark cycles were allowed conventional rat chow diet (0.7% NaCl) and water *ad lib* until the time of the experiment. The rats were anesthetized with pentobarbiturate (70-75 mg/kg, i.p.). As previously described (Berg et al., 2010), the rats were instrumented with a catheter in the femoral artery to monitor systolic (SBP) and diastolic (DBP) BP, and a flow probe on the ascending aorta to register CO and HR. MBP (SBP-DBP/3 + DBP), TPR (MBP/CO), and stroke volume (SV = CO/HR) were calculated. *T*_F_ (time from onset of flow to maximum rise in flow, derived from high resolution aorta flow data, i.e., 5000 points/2 s) was used to indicate changes in inotropy (Berg et al., 2010). The rats were on a positive pressure ventilator throughout the experiment, ventilated with air. Measurements of blood gas parameters demonstrated adequate ventilation in both strains (Berg, 2002, 2003). Body temperature was maintained at 37–38°C by external heating, guided by a thermo sensor inserted inguinally into the abdominal cavity. After completion of the surgery, the arterial catheter was flushed with 0.15 ml PBS (0.01 M Na-phosphate, 0.14 M NaCl, pH 7.4) containing 500 IU heparin/ml. Drugs were dissolved in PBS, and, unless otherwise indicated, administered as bolus injections (0.6–1 ml/kg) through a catheter in the femoral vein, flushed with 0.1 ml PBS.

### Experimental design

To stimulate endogenous release of norepinephrine, all rats were infused for 15 min with tyramine (1.26 μmol/min/kg, 217 μl/min/kg, Berg, 2005). The control group (PBS + tyramine) was injected with PBS 10 min prior to the tyramine-infusion. To identify responses caused by the tyramine-induced reverse transport through NET, rats were injected i.p. with the NET inhibitor desipramine hydrochloride (44 μmol/kg) 5 h prior to the experiment (Miralles et al., 2002; Berg et al., 2012), and pre-treated with PBS 10 min before tyramine during the experiment (desipramine + PBS + tyramine). To test if the tyramine-evoked rise in BP elicited baroreceptor activation and reflex vagal inhibition of HR, another group was pre-treated with the muscarinic receptor antagonist atropine sulfate (2.9 μmol, Berg, 2002) 20 min before tyramine (atropine + tyramine). To analyze the influence of α_2_AR, rats were pre-treated with the non-selective α_2_AR antagonist L-659,066, which does not penetrate the blood-brain barrier (Clineschmidt et al., 1988; 4.4 μmol/kg, 10 min before tyramine, Berg et al., 2012; L-659,066 + tyramine), or with the non-selective, α_2_AR-agonist clonidine, which easily penetrates the blood-brain barrier (151 nmol/kg, 15 min prior to tyramine, Berg et al., 2012). Clonidine was injected 10 min after a sham-injection with PBS (PBS + clonidine + tyramine) or L-659,066 as above (L-659,066 + clonidine + tyramine) to differentiate between involvement of CNS and peripheral α_2_AR. In a time-control group, the rats were pre-treated with PBS and subsequently infused with PBS instead of tyramine (PBS + PBS). The number of rats per group is shown in Table 1.

**Table 1:** **Cardiovascular baselines prior to tyramine and, in parenthesis, the response to pre-treatment**.

Pre-treatment	WKY					SHR				
	N	MBP (mm Hg)	HR (beats/min)	CO (ml/min)	TPR (mm Hg/ml/min)	N	MBP (mm Hg)	HR (beats/min)	CO (ml/min)	TPR (mm Hg/ml/min)
PBS (control)	17	77 ± 4 (3 ± 3)	338 ± 8 (−4 ± 7)	36 ± 2 (4 ± 1)	2.2 ± 0.2 (−0.2 ± 0.1)	15	91 ± 4* (−6 ± 8)	368 ± 8* (−20 ± 7)	17 ± 1* (1 ± 1)	5.4 ± 0.3* (−0.6 ± 0.2)
PBS after desipramine	6	50 ± 2 (−2 ± 3)	310 ± 14 (−4 ± 13)	28 ± 3 (0 ± 1)	1.9 ± 0.2 (−0.1 ± 0.1)	6	63 ± 7† (−8 ± 3)	350 ± 29 (−8 ± 5)	13 ± 2 (−1 ± 0)	4.8 ± 0.3 (−0.2 ± 0.3)
Atropine sulfate	6	63 ± 6 (−8 ± 9)	324 ± 11 (−16 ± 13)	34 ± 2 (4 ± 3)	1.9 ± 0.2 (−0.5 ± 0.2)	6	99 ± 7 (−11 ± 15)	356 ± 5 (−24 ± 8)	19 ± 1 (2 ± 2)	5.1 ± 0.3 (−1.3 ± 0.5)
PBS + clonidine	7	64 ± 2 (−4 ± 5)	316 ± 7 (−35 ± 7)^†^	41 ± 3 (12 ± 1)^†^	1.6 ± 0.1^†^ (−0.8 ± 0.1)^†^	10	56 ± 4^†^ (−42 ± 7)^†^	318 ± 9^†^ (−113 ± 13)^†^	16 ± 1 (−1 ± 1)	3.5 ± 0.2^†^ (−2.2 ± 0.4)^†^
L-659,066	6	50 ± 3^†^ (−16 ± 2)^†^	322 ± 11 (−25 ± 6)	32 ± 3 (1 ± 1)	1.6 ± 0.1 (−0.6 ± 0.0)^†^	7	76 ± 5 (−12 ± 3)	400 ± 14 (−23 ± 10)	19 ± 2 (1 ± 1)	4.3 ± 0.4 (−0.8 ± 0.2)
L-659,066 + clonidine	8	50 ± 2^†^ (−24 ± 2)^†§^	319 ± 7 (−48 ± 8)^†^	32 ± 2 (3 ± 1)	1.6 ± 0.1^†^ (−1.0 ± 0.1)^†§^	7	44 ± 2^†‡§^ (−53 ± 7)^†§^	334 ± 13^§^ (−92 ± 15)^†§^	16 ± 1 (−4 ± 1)^§^	2.8 ± 0.1^†‡§^ (−2.3 ± 0.4)^†§^

Since desipramine was administered before cardiovascular recording was started, its effects were measured by the differences in baselines compared to that in the corresponding PBS-control group. After one-way ANOVA revealed the presence of group differences within each strain (*P* ≤ 0.05), these were located as indicated after comparisons between PBS-control and experimental groups ( †), between groups given L-659,066 + clonidine and PBS + clonidine (‡), and between the L-659,066 + clonidine and L-659066-treated groups (§). Comparison between WKY and SHR was done for the baselines in the PBS + tyramine control groups only (*). Baselines in the PBS + PBS time-control group did not differ from that in the corresponding PBS + tyramine control group (data not shown). *P ≤ 0.05. †, ‡, § – *P* ≤ 0.0071.

### Measurement of plasma catecholamines

Blood was sampled from the femoral artery catheter immediately after the 15-min tyramine-observation period but without discontinuing the infusion of tyramine. About 1.5 ml blood was collected into tubes containing 40 μl 0.2 M glutathione and 0.2 M EGTA. Plasma was stored at −80°C until norepinephrine and epinephrine concentrations were determined with an HPLC-electrochemical detection method (Jensen et al., 2005).

### Drugs

L-659,066 was a kind gift from Merck, Sharp, and Dohme Labs, Rahway, NJ, USA. The remaining drugs were from Sigma Chemical Co., St. Louis, MO, USA.

### Statistical analyses

The results are presented as mean values ± SEM. Data for MBP, HR, CO, SV, and TPR were averaged every minute. The mean of *T*_F_ (indicating changes in inotropy) from five 2-s-period collections of high resolution data was used to determine *T*_F_ at specific times. The cardiovascular response-curves to tyramine were analyzed using Repeated Measures Analyses of Variance and Covariance, first as over-all tests within each strain, and subsequently for each group separately or between groups. Significant responses and group differences were subsequently located using one- and two-sample Student’s *t*-tests, respectively, at specific times. Effects of pre-treatment, differences in cardiovascular baselines, and Δ*T*_F_ were first evaluated by one-way ANOVA including all groups within each strain separately, followed by *post hoc* analyses using two-sample Student’s *t*-tests to locate differences between groups. Significant changes in *T*_F_ (Δ*T*_F_) were detected with one-sample Student’s *t*-tests. Differences in cardiovascular baselines between WKY and SHR were tested in the PBS + tyramine control groups only using two-sample Student’s *t*-tests. Differences in the plasma catecholamine concentrations across groups and strain were first evaluated by two-way ANOVA, followed by two-sample Student’s *t*-tests to locate group differences. In the presence of out-liers, two-sample Student’s *t*-tests were substituted with non-parametric Kruskal–Wallis tests. For all tests and at each step, testing proceeded only when the presence of significant differences and/or interactions were indicated. The *P*-value was for all tests and each step adjusted according to Bonferroni, except for the catecholamine and *T*_F_ data, where *P* ≤ 0.05 was considered significant.

## Results

### Cardiovascular baselines and the response to pre-treatment

MBP, HR, and TPR baselines were higher in SHRs than in WKYs, whereas CO was less (Table 1). The NET inhibitor desipramine reduced MBP in SHRs, but otherwise had no significant effect on baselines (Tables 1 and 2). Changes in baselines were also not detected after pre-treatment with atropine (Tables 1 and 2). As previously described (Berg et al., 2012), clonidine-induced first a peripheral, vascular response, i.e., a transient increase in MBP and TPR, which was reduced by L-659,066 in WKYs and eliminated by L-659,066 in SHRs (data not shown). The subsequent clonidine-induced fall in MBP, TPR, and HR was greater in SHRs than in WKYs also in the presence of L-659,066 (*P* ≤ 0.001), and MBP, HR, and TPR in SHRs after L-659,066 + clonidine no longer differed from that in the WKY controls (*P* = NS; Table 1). L-659,066 alone reduced MBP and TPR baselines in WKYs, but had no significant effect in SHRs. Clonidine and L-659,066 had no effect on inotropy in WKYs (Table 2). However, clonidine had a negative inotropic effect (prolonged *T*_F_) and L-659,066 a minor, positive inotropic effect in SHRs (Table 2).

**Table 2:** **Changes in *T*_F_ as an indication of changes in inotropy in response to pre-treatment and tyramine**.

Pre-treatment	WKY		SHR	
	Δ*T*_F_ (%) Pre-treatment	Δ*T*_F_ (%) Tyramine	Δ*T*_F_ (%) Pre-treatment	Δ*T*_F_ (%) Tyramine
PBS (control)	4 ± 2	−30 ± 1*	4 ± 2	−31 ± 2*
PBS after desipramine	5 ± 2	0 ± 2^†^	5 ± 3	−11 ± 5^†^
Atropine sulfate	−1 ± 1	−28 ± 1*	3 ± 2	−38 ± 2*^†^
PBS + clonidine	2 ± 3	−36 ± 3*	16 ± 6*^†^	−38 ± 4*^†^
L-659,066	2 ± 6	−35 ± 4*	−6 ± 4^†^	−34 ± 1*
L-659,066 + clonidine	11 ± 8	−37 ± 3*^†^	12 ± 11	−30 ± 5*

% Change in *T*_F_ (Δ*T*_F_) indicated differences in inotropy, i.e., a prolongation of *T*_F_ indicated a negative inotropic effect. * – significant changes in Δ*T*_F_. † – significant differences between the control and the experimental groups. *, † – *P* ≤ 0.05.

### The response to tyramine

#### Norepinephrine overflow to plasma

Tyramine greatly increased the plasma concentration of norepinephrine in both strains (*P* < 0.001, PBS + tyramine controls compared to PBS + PBS time-controls), and to a greater extent in SHRs than in WKYs (Table 3). This tyramine-induced overflow was abolished by pre-treatment with the NET inhibitor desipramine in both strains (*P* = NS compared to the PBS + PBS time-controls).

**Table 3:** **The plasma concentration of norepinephrine and epinephrine at the end of the tyramine-infusion period**.

Treatment	WKY		SHR	
	Norepinephrine (nM)	Epinephrine (nM)	Norepinephrine (nM)	Epinephrine (nM)
PBS + PBS (time−control)	0.6 ± 0.1	7.3 ± 1.3	1.4 ± 0.1*	11.9 ± 1.7
PBS + tyramine (control)	27.3 ± 1.1^†^	2.9 ± 0.5^†^	34.1 ± 3.6^†^*	11.2 ± 2.1*
Desipramine + PBS + tyramine	0.9 ± 0.1^‡^	0.4 ± 0.1^‡^	3.1 ± 1.0^‡^	2.2 ± 0.7^‡^
PBS + clonidine + tyramine	27.3 ± 1.6	0.8 ± 0.1^‡^	26.0 ± 1.7^‡^	2.5 ± 1.5^‡^
L−659,066 + tyramine	35.7 ± 2.9^‡^	10.2 ± 3.1^‡^	38.8 ± 5.1	13.6 ± 4.0
L−659,066 + clonidine + tyramine	26.9 ± 1.9^⊣^	9.0 ± 4.1^‡§^	42.1 ± 5.8^§^	22.0 ± 10.2^§^

Plasma was collected at the end of the tyramine-infusion period. Two-way ANOVA demonstrated the presence of significant differences between groups and strain and for group differences across strain (*P* ≤ 0.019). Comparisons were subsequently made between SHRs and WKYs in the PBS + PBS time-control and PBS + tyramine control groups (*), between corresponding PBS + PBS time-control and PBS + tyramine control groups (†), between corresponding PBS + tyramine control and experimental groups (‡), between groups pre-treated with PBS + clonidine + tyramine and L-659,066 + clonidine + tyramine (§), and groups pre-treated with L-659,066 + tyramine and L−659,066 + clonidine + tyramine (⊣). Significant differences were detected as indicated. Plasma catecholamine concentrations were not recorded in the atropine-treated rats. *, †, ‡, §, ⊣ – *P* ≤ 0.05.

The tyramine-induced norepinephrine overflow in WKYs was not different after pre-treatment with clonidine, but was increased after L-659,066. The latter increase was eliminated by additional pre-treatment with clonidine (*P* = 0.049). The opposite pattern was observed in SHRs, where tyramine-stimulated norepinephrine overflow was not different after pre-treatment with L-659,066, but was reduced after clonidine (*P* = 0.006), in fact to the same level as that in the WKY tyramine controls (*P* = NS). This reduction was eliminated when L-659,066 was given prior to clonidine, and the plasma norepinephrine concentration in the SHR L-659,066 + clonidine + tyramine group was not different from that in the SHR PBS + tyramine controls. Since L-659,066 does not cross the blood-brain barrier, these results showed that inhibition of peripheral α_2_AR enhanced tyramine-induced overflow of norepinephrine in WKYs, whereas the elevated overflow in SHRs was not controlled by peripheral α_2_AR.

#### The secretion of epinephrine

We have previously shown that the experiment itself induced some secretion of epinephrine, since the plasma concentration at the end of the experiment in time-controls given PBS + PBS was higher than in rats where blood was collected immediately after femoral artery catheterization and with no other surgery (Berg et al., 2012; Table 3). This secretion was reduced by tyramine in WKYs (*P* = 0.004, PBS + tyramine controls compared to PBS + PBS time-controls), but not in SHRs. The plasma epinephrine concentration was therefore higher in SHRs than in WKYs in the PBS + tyramine controls, but not in the PBS + PBS time-controls. The level of circulating epinephrine was reduced after desipramine + tyramine in both strains.

L-659,066 increased the plasma concentration of epinephrine in WKYs, but not in SHRs, whereas clonidine clearly reduced the concentration of epinephrine in both strains. This decrease was abolished by prior administration of L-659,066 (L-659,066 + clonidine + tyramine); in WKYs returned to the same level as in the L-659,066-only group, i.e., higher than in the WKY controls, and in SHRs, to the same elevated level as in the SHR PBS + tyramine controls.

#### The cardiovascular response

**Figure 2:**
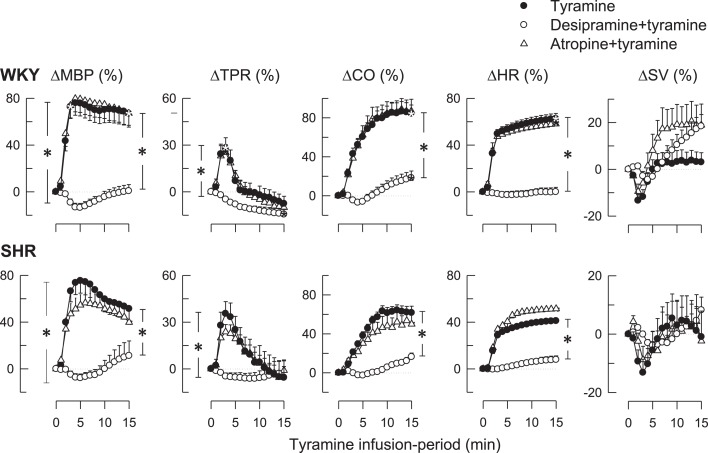
**The effect of the NET inhibitor desipramine and the muscarinic antagonist atropine on the cardiovascular response to tyramine**. After curve evaluation using Repeated Measures Analyses of Variance and Covariance (please see Materials and Methods), significant responses (* within symbol) were detected at 3 and 15 min for ΔMBP, ΔTPR, and ΔSV, and at 15 min for ΔCO and ΔHR, as indicated. Significant group differences at 3 min (* in left brackets) or 15 min (* in right brackets) were detected as indicated. Baselines prior to tyramine are shown in Table 1. *within symbols: *P* ≤ 0.025 for ΔMBP, ΔTPR and ΔSV, and *P* ≤ 0.05 for ΔCO and ΔHR. * in brackets: *P* ≤ 0.001.

As previously documented (Berg, 2005; Berg et al., 2010), tyramine-induced an immediate, but transient rise in TPR and a sustained increase in MBP, CO, and HR (Figure 2). Tyramine also reduced *T*_F_ (Table 2), indicating a positive inotropic response, but had little effect on SV (Figure 2). These responses were all virtually eliminated by desipramine (Figure 2; Table 2), verifying their dependence on NET-mediated norepinephrine release. The HR-response to tyramine was not different after atropine in either strain (Figure 2), although a minor, further decrease in *T*_F_ was observed in SHRs (Table 2).

**Figure 3:**
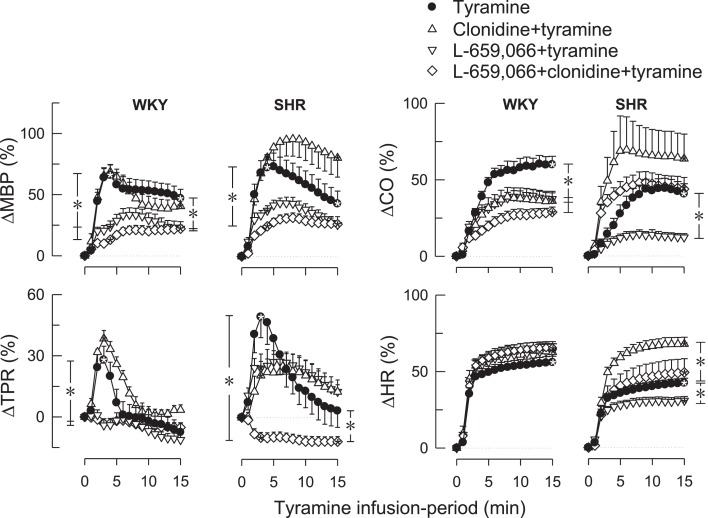
**The changes in MBP, TPR, CO, and HR in WKYs and SHRs during tyramine-induced norepinephrine release after pre-treatment with the centrally active, non-selective α_2_AR-agonist clonidine and the peripherally restricted α_2_AR antagonist L-659,066, separately or combined**. After curve evaluation using Repeated Measures Analyses of Variance and Covariance (please see Materials and Methods), significant responses (* within symbol) and group differences (* in brackets) were detected at 3 and 15 min for ΔMBP and ΔTPR, and at 15 min for ΔCO and ΔHR, as indicated. Baselines prior to tyramine are shown in Table 1. * within symbols and * in brackets: *P* ≤ 0.025 for ΔMBP and ΔTPR, and *P* ≤ 0.05 for ΔCO and ΔHR.

Clonidine alone had no significant effect on the TPR-peak-response to tyramine in either strain (Figure 3). L-659,066 eliminated the TPR-response to tyramine in WKYs (*P* ≤ 0.008 compared to the controls), and this reduction was not different after additional pre-treatment with clonidine. However, in SHRs, L-659,066 alone had no significant effect on the TPR-response to tyramine, whereas L-659,066 + clonidine totally eliminated the tyramine-induced rise in TPR and even precipitated a vasodilatory TPR-response (Figure 3). These changes were paralleled by a lower tyramine-induced rise in MBP after L-659,066 and L-659,066 + clonidine in WKYs and after L-659,066 + clonidine in SHRs (Figure 3). The rise in CO (Figure 3) was reduced in L-659,066- and/or clonidine-treated WKYs, and after L-659,066 in SHRs, paralleled by a reduction in SV (not shown). The tyramine-induced tachycardia was not different in L-659,066 and/or clonidine-treated WKYs, but was reduced after L-659,066 and increased after clonidine in SHRs (Figure 3). L-659,066 + clonidine enhanced the positive inotropic effect of tyramine, i.e., further decreased *T*_F_, in WKYs, whereas clonidine but not L-659,066 + clonidine enhanced the response in SHRs (Table 2).

## Discussion

In the present study, tyramine-induced reverse transport of norepinephrine through NET increased norepinephrine overflow to plasma with a greater effect in SHRs than in WKYs. We demonstrated that pre-treatment with the α_2_AR antagonist L-659,066 enhanced this overflow in WKYs, but had no effect on the elevated overflow in SHRs. The tyramine-activated release of norepinephrine induced a transient rise in TPR, which was totally abolished by L-659,066 in WKYs but not in SHRs. These α_2_AR abnormalities in SHRs were eliminated when the pre-treatment with L-659,066 was combined with clonidine.

Unlike vesicular release, which is Ca^2+^-dependent, NET-mediated norepinephrine release is not under presynaptic control (Starke et al., 1989). Still, inhibition of peripheral α_2_AR increased the tyramine-induced norepinephrine overflow to plasma in WKYs, indicating that presynaptic α_2_AR modified the response. The increase was eliminated after additional pre-treatment with clonidine, further supporting an α_2_AR involvement. Tyramine stimulates reverse transport through NET (Mandela and Ordway, 2006), as confirmed by the fact that desipramine eliminated the tyramine-induced norepinephrine overflow. It therefore seemed that by engaging NET in release, tyramine blocked re-uptake, and, through that, allowed presynaptic control of release to be reflected as differences in norepinephrine overflow to plasma (Figure 1). The condition created by tyramine, therefore appeared to parallel that created by inhibition of NET norepinephrine re-uptake by desipramine, where α_2_AR-mediated inhibition of release was detected as an increase in the plasma norepinephrine concentration in rats where endogenous norepinephrine release was not stimulated (Berg et al., 2012). We therefore concluded that the protocol developed in the present study allow tyramine to be used as a tool to investigate α_2_AR-induced modulation of norepinephrine release.

Tyramine penetrates the blood-brain barrier poorly (Oldendorf, 1971), and its action will therefore be restricted to peripheral nerves. The same is true of L-659,066. The α_2_AR responsible for inhibiting norepinephrine release were therefore likely to be located on peripheral sympathetic nerve terminals. In WKYs, α_2_AR-mediated inhibition of norepinephrine release during tyramine-stimulation appeared to be fully activated, since the plasma norepinephrine concentration was not reduced by clonidine. However, α_2_AR inhibition of release appeared dysfunctional in SHRs, since L-659,066 did not augment norepinephrine overflow in this strain. This conclusion was in agreement with that observed by others (Remie et al., 1992; Zugck et al., 2003). The plasma norepinephrine concentration in the SHR tyramine controls was therefore higher than in WKYs, and similar to that in WKYs pre-treated with α_2_AR antagonist. In SHRs, α_2_AR stimulation with clonidine reduced the plasma norepinephrine concentration and allowed L-659,066 to augment release, i.e., overflow. However, it could not from the present experiments be concluded if the restoring effect of clonidine on the failing α_2_AR control of norepinephrine release in SHRs involved activation of peripheral α_2_AR directly or was due to the reduced central sympathetic output following clonidine in this strain.

We have in previous experiments demonstrated that the level of circulating epinephrine in time-controls exposed to a full experiment, i.e., artificial ventilation, open chest, and flow probe on the ascending aorta, was greater than that in rats where blood was sampled immediately after femoral artery catheterization, without further surgery (Berg et al., 2012). Secretion of epinephrine was therefore induced by the trauma of the experiment, most likely due to surgical stress and activation of the sympatho-adrenal axis. This conclusion was compatible with the observed delay in the rise in circulating epinephrine compared to that of norepinephrine in tyramine-stimulated rats (Berg et al., 2010). Mechanisms that control this trauma-stimulated epinephrine secretion could therefore be examined. Since L-659,066 increased the plasma epinephrine concentration in WKYs but not in SHRs, α_2_AR auto-inhibition of also epinephrine secretion apparently failed in SHRs. Clonidine reduced the plasma concentration of epinephrine in both strains, and this reduction was eliminated after additional pre-treatment with L-659,066. Thus, α_2_AR-mediated inhibition of epinephrine secretion, unlike that of norepinephrine, was not fully activated in WKYs. Moreover, clonidine restored also the failing control of epinephrine secretion in SHRs, similar to that of norepinephrine.

The plasma epinephrine concentration was reduced by tyramine in WKYs. This suggested that epinephrine-producing cells did not contain NET. The tyramine-induced reduction in epinephrine secretion may be explained if tyramine stimulated norepinephrine release in the adrenal glands, and thereby activated α_2_AR on neighboring epinephrine-secreting cells. Activation of α_2_AR inhibition of epinephrine release may also result from the high norepinephrine concentration in plasma due to tyramine-stimulated neuronal release. A similar effect was not observed in SHRs, in agreement with an α_2_AR malfunction of release control in this strain. The contribution of NET located on adrenal norepinephrine-producing cells to the plasma norepinephrine concentration was not fully clarified in the present study. However, preliminary studies indicated that the α_2_AR antagonist L-659,066 increased tyramine-stimulated norepinephrine overflow in WKYs also after acute adrenalectomy (Berg, unpublished observations).

The epinephrine concentration was in both strains reduced by desipramine, a tricyclic antidepressant which enters the CNS. A similar reduction was also seen without tyramine-stimulated norepinephrine release (Berg et al., 2012). This reduction may in WKYs be explained by that desipramine inhibited re-uptake in norepinephrine-secreting adrenal cells and activated close by α_2_AR, which inhibited epinephrine secretion, as discussed above. However, in SHRs, due to the elevated sympathetic tone in this strain, this reduction may be explained by a reduced CNS sympathetic output, since intracerebroventricular application of desipramine reduced BP and CNS norepinephrine overflow to plasma in rabbits (Esler et al., 1995). It is also possible that desipramine through a central action interfered with the trauma-induced activation of epinephrine secretion. The inhibitory effect of desipramine on the secretion of epinephrine as well as its possible central action may explain why desipramine reduced baseline BP in SHRs. In addition, these effects will have limiting implications for the use of desipramine to study the physiology of catecholamine release control.

The peripheral antagonist L-659,066 induced a sustained reduction in TPR baseline in WKYs, demonstrating the presence of a basal α_2_AR-induced vasoconstrictory tone in WKY. The influence of endothelial α_2A_AR-activated NO-synthesis was therefore not detected in the present study, even though NO-dependent vasodilatation dominated the influence of α_2_AR stimulation in the isolated rat aorta (Carter et al., 2002). This is probably explained by the fact that *in vivo* norepinephrine is mainly released abluminally and therefore has access more readily to receptors on the VSMC than on the endothelium. It may be noted that basal release of norepinephrine was also enhanced by L-659,066, as demonstrated in our previous study involving desipramine, but not tyramine (Berg et al., 2012). L-659,066 did not reduce baseline TPR in SHRs, indicating a failing VSMC α_2_AR tone, in agreement with also a reduced sensitivity to the G_i_-inhibitor pertussis toxin in this strain (Berg et al., 2009). α_2_AR-mediated differences in catecholamine release apparently did not influence TPR. However, differences in release may explain the negative and positive inotropic effect of clonidine and L-659,066, respectively.

Peripheral NET-mediated norepinephrine release was responsible for the cardiovascular responses following tyramine, since these were all virtually eliminated by desipramine. α_2_AR-mediated control of TPR during tyramine-stimulation could not be further activated in either strain, since clonidine alone did not enhance the TPR-response to tyramine. This observation paralleled that observed in previous experiments, where clonidine had no effect on the TPR-response to the α_1_AR agonist phenylephrine (Berg et al., 2012). The vasoconstrictory TPR-response to tyramine was totally eliminated after pre-treatment with L-659,066 in WKYs. This reduction, like the L-659,066-dependent fall in TPR baseline, was likely to be explained by an increased adenylyl cyclase-induced vasodilatation, probably activated by βAR-G_s_-signaling, allowed to dominate VSMC tension control when the α_2_AR-G_i_ pathway was blocked (Figure 1). This assumption was supported by the fact that βAR-blockers enhanced the tyramine-induced vasoconstriction (Berg et al., 2010). Thus, in the presence of α_2_AR antagonist, the TPR-response to tyramine in WKYs was low even though plasma norepinephrine and epinephrine levels were increased, underlining the importance of VSMC receptor interaction for the final effect on VSMC tension. However, in SHRs, L-659,066 alone did not inhibit tyramine-induced vasoconstriction, but did so, and even resulted in tyramine-induced vasodilatation, when combined with clonidine. We detected no strain-related difference in the ability of L-659,066 to moderate the α_1_AR-mediated vasoconstrictory response to phenylephrine (Berg et al., 2012). We therefore concluded that similar to that seen for peripheral catecholamine release control, VSMC α_2_AR were dysfunctional in SHRs only when norepinephrine release was activated. Therefore, the failing α_2_AR control of TPR in SHRs, like that of catecholamine release, occurred as a consequence of stimulation of release.

Norepinephrine released by tyramine was likely to be the agonist responsible for presynaptic α_2_AR release modulation, acting in concert with numerous other agonists released from the same or adjacent neurons, or otherwise present in the nearby intercellular space (Kubista and Boehm, 2006; Burnstock and Verkhratsky, 2010; MacArthur et al., 2011). For instance, epinephrine from the circulation or co-released with norepinephrine from sympathetic nerve terminals may activate presynaptic β_2_AR, which stimulate release of norepinephrine (Majewski, 1983), and, in that manner, interfere with α_2_AR inhibition of release. Similarly, a possible co-release of other sympathetic nerve transmitters such as neuropeptide Y and ATP (Burnstock and Verkhratsky, 2010; MacArthur et al., 2011) may also influence the impact of α_2_AR inhibition of release. However, such reactions will not neutralize the observations made with the use of α_2_AR-selective agonist or antagonist, but may explain why a failing α_2_AR inhibition of norepinephrine and epinephrine release was observed during tyramine-stimulated release but not in desipramine-treated SHRs without stimulation of release (Berg et al., 2012). This conclusion was compatible with that α_2_AR inhibition of epinephrine secretion was not impaired in isolated adrenal medulla from SHRs of comparable age (Moura et al., 2011). Also the postsynaptic effect of norepinephrine on VSMC will be influenced by vasoactive agents co-released with or released by norepinephrine (Burnstock and Verkhratsky, 2010; MacArthur et al., 2011). The influence of such interacting agents may be elucidated, one by one, in the same way as we here have studied the contribution of the α_2_AR.

In the present experiments, we measured total norepinephrine overflow and changes in total body vascular resistance. However, a physiological, adrenergic hemodynamic response will be finely regulated to meet situation-dependent and local demands. Still, since tyramine-evoked a general, whole-body sympathetic activation, these gross parameters seemed relevant. Since an important issue of our studies is to gain a better understanding of the etiology of spontaneous hypertension, understanding the mechanisms responsible for sympathetic hyperactivity and the rise in TPR in SHRs, and in essential hypertension in humans is important. The present study design offered an opportunity by which such information may be enhanced.

The rats were connected to a positive pressure ventilator throughout the experiment to maintain stable blood gas values, as previously demonstrated (Berg, 2003). However, positive pressure ventilation will hamper right ventricular ejection, and, hence, reduce venous return to the right atrium, and, through that, left heart function, leading to a reduced CO and consequently BP. We therefore observed a reduction in MBP in SHR (from 127 ± 10 mm Hg before connection to the ventilator to 98 ± 5 mm Hg after completion of surgery and the 10-min stabilization period, *n* = 17, *P* = 0.012), most likely due to the low CO recorded in this strain. A similar reduction in MBP was not observed in WKY (from 75 ± 5 to 74 ± 3 mm Hg, *n* = 13, *P* = NS). In agreement with a weak baroreceptor reflex function in anesthetized rats (Berg et al., 2012), the reduction in MBP baseline in SHR did not result in a strain-related difference in the plasma catecholamine concentrations in time-controls exposed to ventilation and full surgical procedure, or in rats not subjected to artificial ventilation or surgery other than the femoral artery catheterization (Berg et al., 2012). A strain-related difference in un-stimulated plasma norepinephrine overflow was also not seen when re-uptake was blocked by desipramine. A reduction in MBP baseline was not likely to directly influence the tyramine-induced reversed norepinephrine transport through NET in peripheral sympathetic nerves, and, hence, not the cardiovascular responses to tyramine. Activation of baroreceptor reflexes did not influence the cardiovascular response to tyramine, since this was not influenced by atropine. In addition, the cardiovascular responses to tyramine were not compared across strain. We therefore concluded that the low MBP and CO in SHR in the present experimental set-up were not likely to influence our conclusions.

## Conclusion

We concluded that by engaging NET in release, tyramine prevented norepinephrine re-uptake, and, through that, allowed influence of presynaptic control to be demonstrated as differences in norepinephrine overflow to plasma. Tyramine therefore proved a new and useful tool to study receptors involved in presynaptic control of release, in spite of that NET-mediated norepinephrine release itself is not under presynaptic control. In addition, due to the fact that tyramine stimulated endogenous norepinephrine release, influence on a sympathetic cardiovascular response could be studied simultaneously. The adrenal secretion of epinephrine represented a physiological response to the stress induced by the surgical procedure itself. Also this response was sensitive to release control. We were therefore able to demonstrate that peripheral α_2_AR limited both norepinephrine and epinephrine release in WKYs, but failed to do so in SHRs. The same was true for the α_2_AR control of TPR. These disorders in SHRs were observed only when norepinephrine release was stimulated. The α_2_AR malfunctions in SHRs was repaired by the agonist clonidine, either through a direct stimulation of peripheral α_2_AR or through its ability to reduce central sympathetic output. Sympathetic hyperactivity is now generally accepted as a major player in initiating and sustaining hypertension in SHRs and man. α_2_AR auto-inhibition represents an important physiological mechanism to lower adrenergic activity both in CNS and peripherally. The present experiments provided a method by which the mechanisms underlying the failing α_2_AR catecholamines release control in SHRs can be studied *in vivo*. A better understanding of why their function is disturbed in SHRs, may improve our understanding of the pathological mechanisms causing not only spontaneous hypertension, but also other conditions involving α_2_AR, such as diabetes type II and behavior and cognitive disorders (Hunt et al., 1995; Crassous et al., 2007; Fagerholm et al., 2011).

## Conflict of Interest Statement

The authors declare that the research was conducted in the absence of any commercial or financial relationships that could be construed as a potential conflict of interest.
